# Utilization and Spending on Mental Health Services Among Children and Youths With Commercial Insurance

**DOI:** 10.1001/jamanetworkopen.2023.36979

**Published:** 2023-10-03

**Authors:** Mariah M. Kalmin, Jonathan H. Cantor, Dena M. Bravata, Pen-Che Ho, Christopher Whaley, Ryan K. McBain

**Affiliations:** 1 RAND Corporation, Santa Monica, California; 2 Castlight Health, San Francisco, California; 3 Stanford University, Palo Alto, California; 4 RAND Corporation, Arlington, Virginia

## Abstract

This cross-sectional study examines telehealth, in-person, and overall pediatric mental health service utilization and spending rates from January 2019 through August 2022 among a US pediatric population with commercial insurance.

## Introduction

The COVID-19 pandemic severely tested the mental health of children and youths due to unprecedented school closures, social isolation and distancing, and COVID-19–related mortality among family.^1,2^ In response, health systems offered telehealth to increase access to pediatric mental health care.^3^ However, the extent to which telehealth availability led to greater pediatric mental health service utilization and spending is largely unknown. In this study, we examined telehealth, in-person, and overall pediatric mental health service utilization and spending rates from January 2019 through August 2022.

## Methods

In this cross-sectional study among children and youths (aged <19 years) receiving services for the most common pediatric mental health diagnoses (anxiety disorders, adjustment disorder, attention-deficit/hyperactivity disorder [ADHD], major depressive disorder, and conduct disorder), we quantified diagnosis-specific and overall trends and changes in monthly utilization (mental health diagnosis codes used as proxy) and spending rates between 3 phases related to SARS-CoV-2: (1) prepandemic, before the national public health emergency declaration (January 1, 2019, to March 12, 2020); (2) acute, before vaccine availability (March 13 to December 17, 2020); and (3) postacute (December 18, 2020, to August 31, 2022). Monthly medical claims data (categorized by *International Statistical Classification of Diseases and Related Health Problems, Tenth Revision [ICD-10]* diagnostic codes^4^) provided by Castlight Health were used to measure trends in utilization per 1000 beneficiaries and spending (accounting for inflation by indexing 2020 to 2022 rates to 2019) per 10 000 beneficiaries among approximately 1.9 million children and youths with commercial insurance throughout the US (eAppendix in Supplement 1). The RAND institutional review board deemed this study exempt and waived informed consent because deidentified claims data were used. We followed the STROBE reporting guideline.

We estimated longitudinal, fixed-effects regressions segmented by each period for each diagnosis and overall. Fixed effects were included for US state and patient biological sex to account for associated variability. Standard errors were clustered at the state level to account for multiple facilities within each state. Precision estimates were reported using 2-sided 95% CIs. Analyses were conducted with Stata version 16.0 (StataCorp) from April to May 2023.

## Results

Among approximately 1.9 million claims for children and youths with commercial insurance, utilization and spending trends were generally consistent across pediatric mental health diagnoses (Figure), allowing for collapsing of estimates. Compared with prepandemic, in-person pediatric mental health services declined by 42% during the pandemic’s acute phase, while pediatric telehealth services increased 30-fold (3027%), representing a 13% relative increase in overall utilization. By August 2022, in-person services returned to 75% of prepandemic levels and tele–mental health utilization was 2300% higher than prepandemic levels. During the postacute period, we observed a gradual increase in spending rates compared with prepandemic for in-person, telehealth, and total visits. From January 2019 to August 2022, mental health service utilization increased by 21.7%, while mental health spending rates increased by 26.1%.

**Figure.**
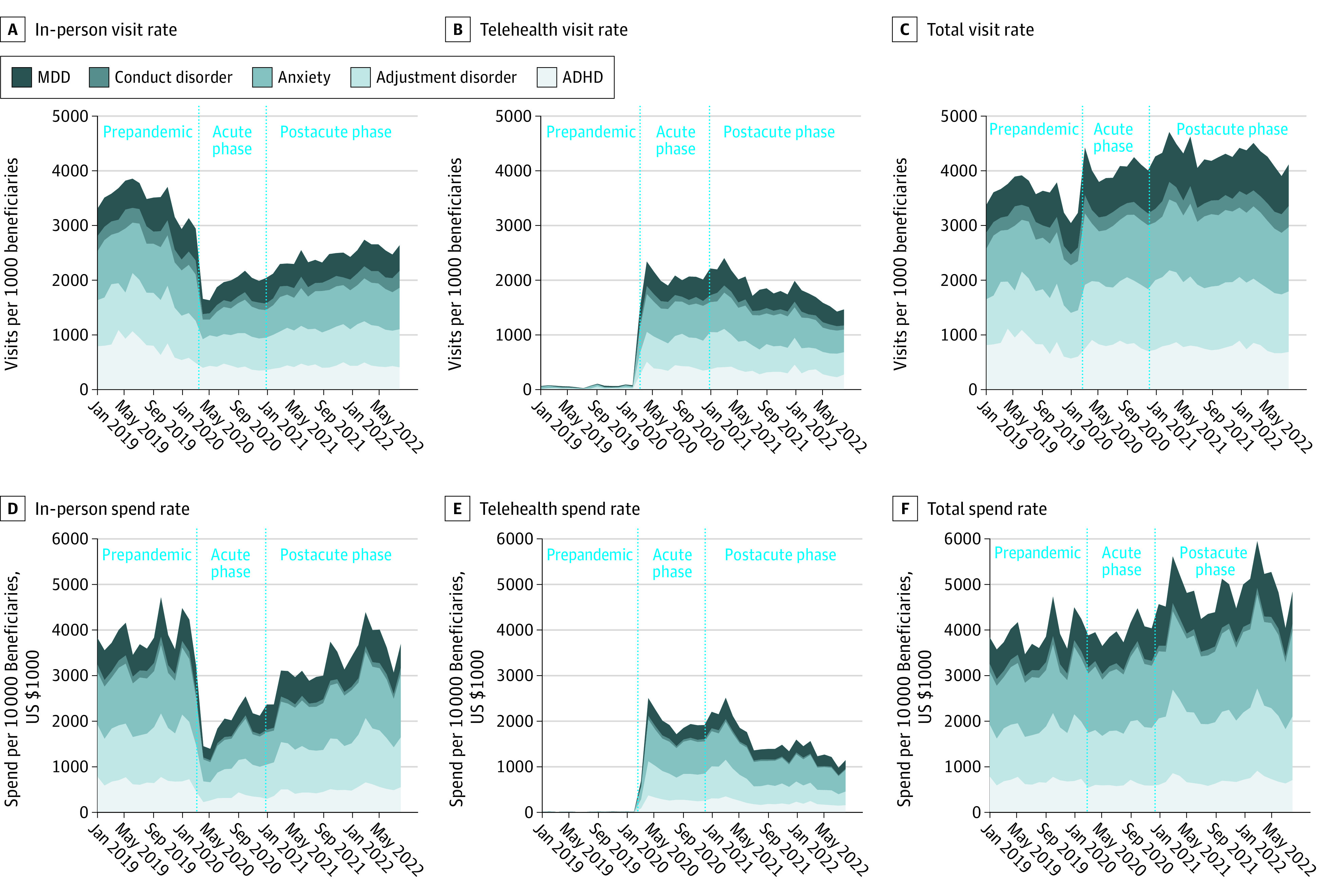
Pediatric In-Person, Telehealth, and Total Mental Health Care Visits and Spending Between January 2019 and August 2022 The figure is a stacked line graph, conveying cumulative visit rates and spend rates across mental health diagnoses.

The Table shows the diagnosis-specific and overall results of the longitudinal, fixed-effects segmented regressions for utilization and spending accounting for state and patient sex among in-person and telehealth visits. For each diagnosis and overall, there was at least 1 statistically significant difference between 2 consecutive periods (intercept term) and at least 1 statistically significant change within each period (slope) for both utilization and spending.

**Table.** Changes in Pediatric In-Person and Telehealth Utilization and Spending Between January 2019 and August 2022^a^

Diagnosis	Prepandemic phase				Acute phase				Postacute phase			
	In-person		Telehealth		In-person		Telehealth		In-person		Telehealth	
	Coefficient (95% CI)	*P* value	Coefficient (95% CI)	*P* value	Coefficient (95% CI)	*P* value	Coefficient (95% CI)	*P* value	Coefficient (95% CI)	*P* value	Coefficient (95% CI)	*P* value
**Major depressive disorder**												
Utilization												
Intercept	2.73 (−32.58 to 38.05)	.88	9.29 (−16.12 to 34.70)	.47	−1.18 (−1.55 to −0.80)	<.001	1.23 (0.60 to 1.86)	<.001	−0.99 (−1.39 to −0.59)	<.001	1.28 (0.63 to 1.93)	<.001
Slope	0.01 (−0.04 to 0.06)	.80	−0.01 (−0.04 to 0.03)	.75	−0.03 (−0.08 to 0.02)	.19	−0.01 (−0.04 to 0.02)	.53	0.00 (−0.04 to 0.04)	.93	−0.04 (−0.07 to −0.01)	.02
Spending												
Intercept	−12 195 (−86 005 to 61 614)	.74	29 994 (−32 894 to 92 881)	.34	−2760 (−3816 to −1704)	<.001	3937 (2786 to 5087)	<.001	−897.50 (−2283 to 488.40)	.20	4130 (2763 to 5498)	<.001
Slope	29.91 (−73.30 to 133.10)	.56	−38.69 (−126.50 to 49.12)	.38	−2.00 (−121.50 to 117.50)	.97	53.47 (−22.84 to 129.80)	.17	33.41 (−14.79 to 81.61)	.17	−93.42 (124.20 to −62.62)	<.001
**Conduct disorder**												
Utilization												
Intercept	28.02 (15.46 to 40.57)	<.001	13.93 (−11.12 to 38.98)	.27	−0.26 (−0.45 to −0.05)	.01	0.02 (−0.15 to 0.19)	.80	−0.33 (−0.58 to −0.09)	.008	−0.05 (−0.25 to 0.15)	.60
Slope	−0.03 (−0.05 to −0.01)	.001	−0.01 (−0.05 to 0.03)	.52	−0.01 (−0.03 to 0.01)	.31	−0.00 (−0.01 to 0.01)	.81	−0.01 (−0.03 to 0.01)	.44	−0.00 (−0.01 to 0.01)	.70
Spending												
Intercept	3232 (−31 505 to 37 969)	.85	−20 796 (−46 679 to 5087)	.11	−946.70 (−1352.00 to −541.10)	<.001	580.30 (286.00 to 874.60)	<.001	−871.00 (−1368.00 to −374.10)	<.001	601.60 (227.50 to 975.80)	.002
Slope	−1046.00 (−49.57 to 47.48)	.97	31.81 (−4.37 to 67.99)	.08	4.32 (−37.74 to 46.37)	.84	−5.15 (−36.01 to 25.71)	.74	5.24 (−15.31 to 25.80)	.61	−26.92 (−40.07 to −13.76)	<.001
**Anxiety disorders**												
Utilization												
Intercept	30.29 (−46.47 to 107.00)	.43	49.76 (−8.38 to 107.90)	.09	−2.53 (−3.17 to −1.88)	<.001	3.07 (1.63 to 4.50)	<.001	−2.46 (−3.09 to −1.83)	<.001	3.11 (1.70 to 4.52)	<.001
Slope	−0.03 (−0.14 to 0.08)	.57	−0.06 (−0.15 to 0.02)	.13	−0.06 (−0.09 to −0.02)	.001	0.01 (−0.04 to 0.07)	.62	0.09 (0.01 to 0.17)	.04	−0.08 (−0.14 to −0.03)	.005
Spending												
Intercept	−119 261 (−213 545 to −24 976)	.01	49 936 (−54 603 to 154 475)	.34	−6989 (−8656 to 5323)	<.001	8444 (5976 to 10 911)	<.001	−6235 (−8159 to −4310)	<.001	8462 (5897 to 11 207)	<.001
Slope	189.40 (57.71 to 321.00)	.006	−62.46 (−209.60 to 84.72)	.40	−0.13 (−131.50 to 321.00)	1.00	120.50 (23.35 to 217.70)	.02	314.30 (−131.50 to 131.20)	<.001	−182.40 (−242.60 to −122.20)	<.001
**Adjustment disorder**												
Utilization												
Intercept	42.58 (−22.47 to 107.60)	.19	−23.45 (−75.76 to 28.86)	.37	−2.13 (−2.75 to −1.50)	<.001	2.11 (0.83 to 3.40)	.002	−1.97 (−2.63 to −1.31)	<.001	2.08 (0.80 to 3.37)	.002
Slope	−0.05 (−0.14 to 0.04)	.31	0.04 (−0.03 to 0.11)	.29	−0.04 (−0.09 to 0.02)	.31	0.01 (−0.04 to 0.06)	.29	0.03 (−0.03 to 0.09)	.35	−0.08 (−0.14 to −0.03)	.004
Spending												
Intercept	−96 838 (−210 917 to 17 242)	.09	−11 094 (−84 827 to 62 639)	.76	−6642 (−8545 to −4739)	<.001	6875 (5168 to 8582)	<.001	−5162 (−6956 to −3368)	<.001	7319 (5578 to 9059)	<.001
Slope	168.10 (8.46 to 327.80)	.04	27.94 (−75.48 to 131.40)	.59	57.64 (−71.74 to 187.00)	.38	63.52 (−38.72 to 165.80)	.22	167.70 (91.91 to 243.60)	<.001	−225.40 (−294.00 to −156.70)	<.001
**Attention-deficit/hyperactivity disorder**												
Utilization												
Intercept	89.66 (−24.06 to 203.40)	.12	29.50 (6.14 to 52.85)	.01	−1.73 (−2.98 to −0.48)	.01	1.22 (0.50 to 1.94)	.001	−1.91 (−3.32 to −0.49)	.009	1.08 (0.34 to 1.81)	.005
Slope	−0.12 (−0.27 to 0.043)	.15	−0.03 (−0.07 to 0.00)	.04	0.04 (−0.13 to 0.22)	.61	0.00 (−0.02 to 0.02)	.99	0.02 (−0.01 to 0.05)	.14	−0.02 (−0.03 to −0.00)	.03
Spending												
Intercept	−10 334 (−58 862 to 38 194)	.67	12 773 (−66 988 to 92 533)	.75	−3842 (−4620 to −3064)	<.001	3404 (1795 to 5013)	<.001	−3647 (−4601 to −2693)	<.001	3557 (1829 to 5285)	<.001
Slope	30.19 (−37.48 to 97.85)	.37	−11.94 (−123.40 to 99.51)	.83	52.10 (−35.47 to 139.70)	.24	15.40 (−82.41 to 113.20)	.75	118.00 (84.56 to 151.50)	<.001	−74.88 (−106.4 to −43.36)	<.001
**Total across all diagnoses**												
Utilization												
Intercept	45.19 (−8.78 to 99.16)	.10	25.49 (−2.12 to 53.11)	.07	−1.58 (−1.99 to 1.18)	<.001	1.57 (0.83 to 2.32)	<.001	−1.57 (−2.03 to −1.11)	<.001	1.53 (0.80 to 2.26)	<.001
Slope	−0.05 (−0.13 to 0.02)	.17	−0.03 (−0.07 to 0.01)	.15	−0.02 (−0.06 to 0.03)	.43	0.00 (−0.03 to 0.03)	.83	0.03 (−0.02 to 0.07)	.24	−0.05 (−0.08 to −0.01)	.008
Spending												
Intercept	−42 880 (−81 362 to −4398)	.03	33 172 (−17 011 to 83 355)	.19	−4164 (−4977 to −3352)	<.001	4605 (3413 to 5797)	<.001	−3306 (−4098 to −2514)	<.001	4732 (3495 to 5970)	<.001
Slope	79.09 (25.42 to 132.80)	.005	−38.88 (−109.50 to 31.74)	.27	24.79 (−43.62 to 93.20)	.47	57.30 (3.95 to 110.60)	.04	124.20 (83.45 to 164.90)	<.001	−124.70 (−154.60 to −94.82)	<.001

^a^ Each column and row combination is a separate longitudinal fixed-effects regression model controlling for US state and sex of the patient. Standard errors were clustered at the state level. Intercept terms indicate change in utilization and/or spending from the previous period, whereas slopes indicate rate of change within each period. Spending rates in 2020 to 2022 were indexed to 2019 to account for inflation. Segment points coincided with the date cutoffs for the 3 periods: prepandemic phase (January 1, 2019, to March 12, 2020), acute phase (March 13 to December 17, 2020), and postacute phase (December 18, 2020, to August 31, 2022).

## Discussion

After comparing mental health care service utilization and spending rates for children and youths with commercial insurance across 3 periods, we found differences between periods as well as different rates of change within each period for both visit types, even after accounting for state and patient sex. Utilization and spending increased over the entire timeframe. ADHD, anxiety disorders, and adjustment disorder accounted for most visits and spending in all phases.

The study has limitations. First, these data represent only children and youths with commercial insurance. Utilization patterns, care needs, and spending may differ for other pediatric patient populations such as Children’s Health Insurance Program recipients or children and youths lacking health insurance. Additionally, we did not have available data to distinguish between new and existing pediatric patients, and thus cannot specify whether increases result from an overall population increase in mental health diagnoses or a utilization increase among existing patients.

Our findings indicate that pediatric telehealth care for mental health needs filled a critical deficit in the immediate period following the emergence of COVID-19 and continues to account for a substantial proportion of pediatric mental health service utilization and spending. Supported by evidence that telehealth can effectively deliver mental health treatment for children and youths,^5,6^ these findings have important implications for telehealth sustainability beyond the effects of COVID-19.

## Notes

Supplement 1. **eAppendix.** Online-Only Supplemental Material

Supplement 2.
Data Sharing Statement
