# Supplementary material for: Utilization and Spending on Mental Health Services Among Children and Youths With Commercial Insurance
Source: JAMA Netw Open. 2023 Oct 3;6(10):e2336979. doi: 10.1001/jamanetworkopen.2023.36979 (PMC10548294; doi:10.1001/jamanetworkopen.2023.36979)
Supplement: Supplement 1. — eAppendix. Online-Only Supplemental Material [file jamanetwopen-e2336979-s001.pdf]

Supplemental Online Content

Kalmin MM, Cantor JH, Bravata DM, Ho P, Whaley C, McBain RK. Utilization and spending on mental health services among children and youths with commercial insurance, 2019-2022. *JAMA Netw Open*. 2023;6(10):e2336979. doi:10.1001/jamanetworkopen.2023.36979

eAppendix. Online-Only Supplemental Material

This supplemental material has been provided by the authors to give readers additional information about their work.

© 2023 Kalmin MM et al. *JAMA Network Open.*

eAppendix. Online-Only Supplemental Material

Medical claims were classified according to the International Statistical Classification of Diseases and Related Health Problems, Tenth Revision (ICD-10) primary diagnosis codes for mental disorders at the 3- and 4-digit level to preserve sufficient cell size for anonymity. These included major depressive disorder (F32, F33), anxiety disorders (F40, F41), bipolar disorder (F31), adjustment disorder (F43.2), and PTSD (F43.1).

© 2023 Kalmin MM et al. *JAMA Network Open.*
