# Supplementary material for: Utilization and Spending on Mental Health Services Among Children and Youths With Commercial Insurance
Source: JAMA Netw Open. 2023 Oct 3;6(10):e2336979. doi: 10.1001/jamanetworkopen.2023.36979 (PMC10548294; doi:10.1001/jamanetworkopen.2023.36979)
Supplement: Supplement 2. — Data Sharing Statement [file jamanetwopen-e2336979-s002.pdf]

Data Sharing Statement

Kalmin. Utilization and Spending on Mental Health Services Among Children and Youths With Commercial Insurance, 2019-2022. *JAMA Netw Open*. Published October 03, 2023. doi:10.1001/jamanetworkopen.2023.36979

Data
Data available: No
